# Competitive Anxiety, Sports Injury, and Playing Category in Youth Soccer Players

**DOI:** 10.3390/children12081094

**Published:** 2025-08-20

**Authors:** Rafael Sánchez-Ruiz, Laura Gil-Caselles, Alejo García-Naveira, Félix Arbinaga, Roberto Ruiz-Barquín, Aurelio Olmedilla-Zafra

**Affiliations:** 1 Department Psychology, University of Murcia, 30100 Murcia, Spain; rafael.sanchezr@um.es; 2 Research Group HUMSE, Faculty of Sports Sciences, University of Murcia, 30100 Murcia, Spain; 3 Department Psychology, University Villanueva, 28034 Madrid, Spain; 4 Department of Clinical and Experimental Psychology, University of Huelva, 21071 Huelva, Spain; felix.arbinaga@dpsi.uhu.es; 5 Interfaculty Department of Developmental and Educational Psychology, Faculty of Teacher Training, University of Autónoma Madrid, 28049 Madrid, Spain; roberto.ruiz@uam.es; 6 Department of Personality, Evaluation, and Psychological Treatment, Faculty of Sports Sciences, University of Murcia, 30100 Murcia, Spain; olmedilla@um.es

**Keywords:** psychological risk factors, injury vulnerability, youth soccer, developmental stages, performance anxiety

## Abstract

Background: Adolescence is a critical period of physical, psychological, and social development, during which athletes are particularly vulnerable to stress and injuries. Competitive anxiety has been identified as a psychological factor that may increase injury risk; however, its role among young soccer players remains underexplored. Objectives: This study aimed to analyse the association between competitive anxiety and injury vulnerability in young male soccer players aged 10 to 15 years. Methods: A total of 322 male soccer players from youth categories (Alevin, Infantil, and Cadete) participated. Competitive anxiety was assessed using the Sport Anxiety Scale-2 (SAS-2), and injury data were collected via a self-reported questionnaire covering the 2024–2025 season. Descriptive, comparative, and correlational analyses were conducted using non-parametric tests. Results: A high incidence of injuries was observed, increasing progressively with age category. In the overall sample, injuries were associated with higher levels of Somatic Anxiety, as well as with age and sporting experience, variables also linked to increased Worry and reduced Distraction. When analysed by category, no significant associations between anxiety and injury were found in Alevin players. In the Infantil group, injury incidence showed a slight increase with age and experience, but no association with anxiety was detected. Among Cadete players, injuries were positively related to Somatic Anxiety and Distraction, highlighting the influence of psychological factors at this developmental stage. Conclusions: These findings underscore the relevance of competitive anxiety, particularly Worry and Distraction, as risk factors for injury in youth soccer. The implementation of preventive psychological interventions and ongoing monitoring is recommended to reduce anxiety levels and injury vulnerability, thereby promoting safer and healthier athletic development among young soccer players.

## 1. Introduction

Adolescence is a period of hormonal, psychological, and social transformation, characterised by increased sensitivity and vulnerability, during which context and lived experiences can significantly shape psychological well-being [1,2,3]. Recent research has highlighted that engaging in sports during this life stage not only promotes physical and mental health but also enhances self-esteem and self-efficacy, strengthens social relationships, improves academic performance, and serves as a protective factor against risky behaviours such as alcohol, tobacco, and drug use [4,5,6].

However, alongside these benefits, sports participation also carries a risk of injury, particularly during early stages of development, where injuries can lead to sport dropout, reduced well-being, or negative psychological consequences such as fear, frustration, anxiety, or loss of athletic identity [7,8,9]. If not properly addressed through preventive, multidimensional approaches adapted to the characteristics of young athletes, these consequences may persist in the medium to long term.

In Spain, soccer stands out not only due to its high participation rate among youths, with over 390,000 boys and girls aged 10 to 15 [10], but also because it is one of the sports with the highest injury incidence during the developmental stages. Studies such as López-Valenciano et al. [11] report 8.1 injuries per 1000 h of play among professional soccer players. In younger populations, injury rates range from 34% to 65% [9]. The most frequent injuries involve the lower limbs, particularly sprains and strains affecting the ankle and knee joints, as well as muscular injuries in the hamstrings and quadriceps [12,13]. The recent literature has also identified several relevant risk factors for injury in young athletes, including competitive level, weekly training volume, cumulative workload, physical and mental fatigue, injury history, and psychosocial stressors within the sporting environment [14,15,16].

Among these, competitive trait anxiety has been identified as one of the psychological constructs with the greatest impact on both performance and injury risk [17,18,19]. It is defined as a general emotional response to perceived threats in evaluative or competitive situations, and includes cognitive (e.g., worries about performance or fear of failure), physiological (e.g., muscle tension, increased heart rate), and behavioural responses (e.g., technical errors or avoidance behaviours) [20,21]. These three components interact in a way that may increase injury likelihood by impairing attention, increasing muscular stiffness, and altering coordination, thus facilitating errors or improper movements during performance [22,23,24].

Among youth soccer players, the relationship between high anxiety levels and injury occurrence has been consistently observed [25,26,27]. In the Spanish context, Berengüí [28] and Berengüí and Puga [29] have underlined the predictive role of anxiety and other psychological variables in sports injuries. Competitive anxiety tends to increase when young athletes perceive high external demands and lack effective coping resources or emotional support. Furthermore, the Stress and Injury Model by Andersen and Williams [30,31] proposes that personal traits, life stressors, and coping skills influence athletes’ responses to stress and injury susceptibility.

In this context, the Sport Anxiety Scale-2 (SAS-2) [32] has been widely used as a valid and reliable tool for assessing competitive trait anxiety in young athletes. Previous research has reported associations between elevated levels of somatic anxiety, worry, and concentration disruption with increased injury risk, poor performance, and even sport withdrawal [27,33,34,35,36]. These findings have been observed across multiple disciplines, including soccer [37] and water polo [36], supporting the scale’s validity and utility in youth sport settings.

Moreover, sport age category may play a critical role in the development and manifestation of competitive anxiety, as psychological demands and developmental milestones vary across stages such as Alevin (10–11 years), Infantil (12–13 years), and Cadete (14–15 years) [37,38,39,40]. Each category is associated with distinct challenges regarding biological maturation, training loads, competitive pressure, and external expectations, which may influence how young athletes perceive threat and apply coping strategies [41,42,43]

Although some studies have explored the relationship between psychological variables and injury risk in young athletes, most have adopted cross-sectional designs and focused on adult or elite populations. Longitudinal studies that directly examine the influence of competitive trait anxiety on injury occurrence across different developmental categories in youth soccer remain scarce.

Therefore, the aim of this study was to examine the association between competitive trait anxiety and the occurrence of sports injuries over the course of a season in young male soccer players aged 10 to 15 years, while also exploring potential differences based on sport age category.

We hypothesised that players with higher levels of somatic anxiety, worry, and concentration disruption would show greater injury incidence throughout the season. Furthermore, we expected these associations to vary depending on the specific sport age category (Alevin, Infantil, and Cadete), consistent with the developmental and contextual demands of each stage.

## 2. Materials and Methods

### 2.1. Participants

The sample comprised 322 male youth soccer players from Real Murcia C.F. [44] and its affiliated club, Murcia Promises [45], aged between 10 and 15 years (mean [M] = 12.95; standard deviation [SD] = 1.65). The participants were grouped into three sport age categories: Alevin (10–11 years; *n* = 72), Infantil (12–13 years; *n* = 115), and Cadete (14–15 years; *n* = 135). Their mean height was 1.59 m (SD = 0.12) and mean weight was 48.3 kg (SD = 9.8), with no significant differences across categories.

Participants were selected using a non-random, convenience-based sampling method, based on accessibility criteria [46]. The inclusion criteria were as follows: (a) being a registered player in the Alevin, Infantil, or Cadete categories of the selected clubs; (b) being between 10 and 15 years of age; and (c) having a signed informed consent form from a parent or legal guardian.

A total of 24 participants were excluded from the initial pool (*n* = 346): 15 due to the absence of consent, 3 for being female (to avoid biological and psychosocial bias), and 6 for not meeting the age requirements. The final sample thus consisted of 322 players.

### 2.2. Instruments

A bespoke sociodemographic questionnaire was administered to collect data on age, sex, category, club, years of soccer practice, and date of completion.

Competitive anxiety was assessed using the Sport Anxiety Scale-2 (SAS-2) [32], in its Spanish validated version by Ramis et al. [22]. The scale consists of 15 items rated on a 4-point Likert scale (1 = not at all, 4 = very much), distributed across three subscales (5 items each), with good internal consistency: Somatic Anxiety (α = 0.83), Worry (α = 0.78), and Concentration Disruption (α = 0.73).

Somatic Anxiety assesses physiological symptoms associated with anxiety, such as muscle tension, palpitations or stomach discomfort (e.g., “My body feels tense”); Worry evaluates anticipatory negative thoughts related to performance, fear of failure and external evaluation (e.g., “I worry about making mistakes”); and Concentration Disruption refers to difficulty maintaining attention and focus during competition (e.g., “I find it hard to concentrate on what I am doing”).

For the present sample, good reliability levels were obtained for the Somatic Anxiety (α = 0.80) and Worry (α = 0.85) subscales, as well as for the overall questionnaire or Total Anxiety Score (α = 0.83). The Concentration Disruption factor showed moderate reliability (α = 0.66), below the 0.70 threshold. Therefore, a Total SAS-2 Score (sum of the 15 items, range 15–60) and specific scores for each subscale (range 5–20) were calculated, where higher scores indicate greater anxiety [22].

For the present study, a self-report injury questionnaire based on Prieto et al. [33] was administered. Although the instrument allows for the collection of multiple variables related to sports injuries (type and severity), only the total number of injuries sustained during the season was recorded, following the methodological considerations of Van Mechelen et al. [47] and Olmedilla et al. [48].

### 2.3. Procedure

Data collection was carried out in collaboration with the aforementioned clubs during the 2023/2024 season, selected for their regional representativeness and competitive homogeneity. Initial contact was made through the youth soccer coordinator of the club, who facilitated communication with the coaches. Subsequently, families were invited to attend an informative meeting for each team before training sessions, during which the aims and procedures of the study were explained and a QR code was provided granting access to the digital form, which included the information sheet and informed consent form (September 2023).

Once consent had been obtained, the players were scheduled to attend 30 min prior to their regular training in a designated room within the sports facilities that met appropriate conditions (lighting, temperature and noise). There, they completed the questionnaires in groups (each team separately) using their mobile devices via Google Forms, which included a section for informed assent (September 2023). The monitoring and recording of sports injuries throughout the season were carried out by the research team in collaboration with the coaching staff and medical services of the club (from October 2023 to May 2024).

The study was approved by the Ethics Committee of the University of Murcia (CEI-M10/2024/501) and conducted in accordance with the principles of the Declaration of Helsinki [49,50,51], also complying with the Ethical Standards in Sport and Exercise Science Research [52]. Participation was informed, anonymous, and voluntary. The entire process was supervised by the research team.

### 2.4. Design

A non-experimental, observational design was employed, incorporating a cross-sectional approach for the SAS-2 test and a longitudinal approach for the injury register, with a descriptive–correlational nature. According to Montero and León [53], it constitutes an empirical study based on probabilistic surveys.

### 2.5. Data Analysis

For statistical analyses, SPSS version 28.0 (IBM Corp., Armonk, NY, USA) was used. The following analyses were performed: measures of central tendency (means), measures of dispersion (standard deviations), normality tests using the Kolmogorov–Smirnov test, mean difference analyses using the Mann–Whitney U test and the Kruskal–Wallis test, effect size calculation using Hedges’ Ĝ, and correlational analyses using Spearman’s Rho (for quantitative scores) and Kendall’s Tau-b (for ordinal variables, such as number of injuries). A significance level of *p* < 0.05 was adopted for all statistical tests.

## 3. Results

The normality analysis conducted on the total sample revealed that the assumptions of normality were not met for the three SAS-2 factors (Somatic Anxiety, Worry, and Concentration Disruption), as well as for the total score. Specifically, the following results were obtained: Somatic Anxiety (K–S = 0.243; *p* < 0.001), Worry (K–S = 0.080; *p* < 0.001), Concentration Disruption (K–S = 0.151; *p* < 0.001), and Total SAS-2 Score (K–S = 0.096; *p* < 0.001). This non-normality pattern was also observed when analysing the sports age categories (Alevin, Infantil, and Cadete). Only the Total SAS-2 Score in the Alevin category showed a normal distribution (K–S = 0.075; *p* = 0.200; *p* > 0.05).

Regarding the number of injuries, the mean number of injuries was 1.17 (SD = 1.20; n = 322), with a minimum of zero and a maximum of eight injuries. Table 1 shows that 108 players (33.5%) did not suffer any injury, 106 (32.9%) sustained one injury, 76 (23.6%) sustained two injuries, 18 (5.6%) sustained three injuries, 9 (2.8%) sustained four injuries, 2 (0.6%) sustained five injuries, 2 (0.6%) sustained six injuries, and 1 (0.3%) sustained eight injuries. These data indicate that 66.5% of the players experienced at least one sports injury throughout the season.

**Figure 1:**
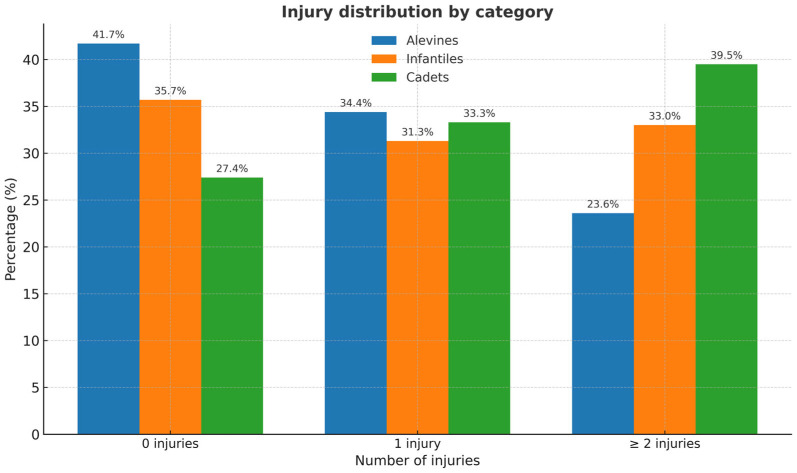
Injury distribution by sports age category.

Additionally, Figure 1 shows that as the sports age category increases, the percentage of players who did not suffer any injury during the season decreases: Alevin (41.7%), Infantil (35.7%), and Cadete (27.4%). A relatively stable trend was observed among the categories in players with one injury: Alevin (34.4%), Infantil (31.3%), and Cadete (33.3%). Lastly, as the sports age category increases, the percentage of players with two or more injuries also increases: Alevin (n = 17; 23.6%), Infantil (n = 38; 33.0%), and Cadete (n = 53; 39.5%).

Regarding the SAS-2 factors and sporting age categories, Table 2 shows that, for the Worry factor, the Cadete category presents the highest scores, with the Infantil category showing very similar values. The Alevin category reports the lowest scores. The application of the Kruskal–Wallis test indicates a statistically significant difference between the categories (*p* < 0.001). Post hoc analyses using pairwise Mann–Whitney U tests reveal differences between Alevin and Infantil (*p* < 0.05; Hedges Ĝ = 0.380) and between Alevin and Cadete (*p* < 0.01; Hedges Ĝ = 0.420), both with a medium effect size. No significant differences are found between Infantil and Cadete.

With regard to the Concentration Disruption factor, Cadete and Infantil players report the lowest and most similar scores, while Alevin players show the highest scores. Post hoc analyses (using pairwise Mann–Whitney U tests) indicate significant differences between Alevin and Infantil (*p* < 0.001; Hedges Ĝ = 0.508) and between Alevin and Cadete (*p* < 0.001; Hedges Ĝ = 0.477), both with a medium effect size. No differences are found between Infantil and Cadete.

Considering the Total SAS-2 Score, the three categories show very similar values, although the highest scores are observed in the Infantil category, followed by Cadete and Alevin. In this case, no statistically significant differences are observed.

**Figure 2:**
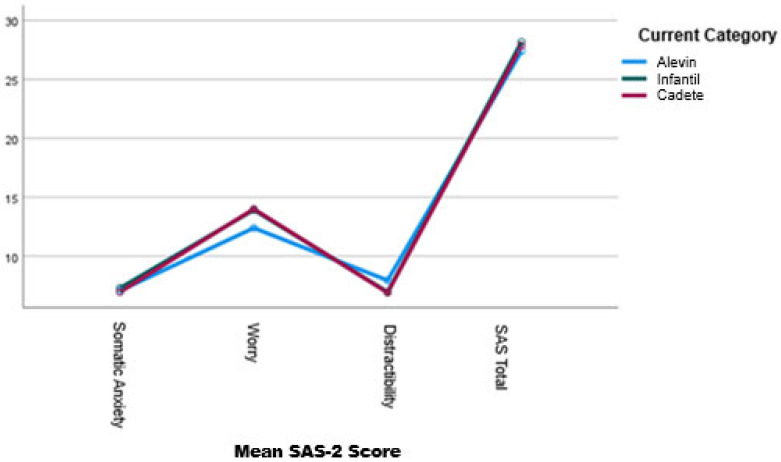
Mean scores of the SAS questionnaire (three factors and total score) according to sports age category.

Figure 2 illustrates how the differences between sporting age categories are mainly reflected in the Worry and Concentration Disruption factors.

Regarding the relationship between the SAS-2 factors, the number of injuries, age, and years of experience for the overall sample of players, the results in Table 3 show positive correlations between the number of injuries and somatic anxiety (Tau-b = 0.091; *p* < 0.05), although the strength of this correlation is low. However, positive correlations are found with age (Tau-b = 0.146; *p* < 0.001) and years of experience (Tau-b = 0.135; *p* < 0.01).

The Somatic Anxiety factor showed moderate positive correlations with Worry (Rho = 0.281, *p* < 0.01) and Concentration Disruption (Rho = 0.380, *p* < 0.01), as well as a strong correlation with Total Anxiety (Rho = 0.611, *p* < 0.01).

With respect to the Worry factor, a strong positive correlation was identified with Total Anxiety (Rho = 0.856, *p* < 0.01), a moderate positive correlation with Concentration Disruption (Rho = 0.225, *p* < 0.01), a weak positive correlation with age (Rho = 0.140, *p* < 0.05), and a trend-level correlation with years of experience (Rho = 0.094, † *p* < 0.10).

As for the Concentration Disruption factor, a moderate positive correlation was found with Total Anxiety (Rho = 0.580, *p* < 0.01) and negative correlations with years of experience (Rho = −0.200, *p* < 0.01) and age (Rho = −0.229, *p* < 0.01).

Total Anxiety was positively correlated with all of its factors (Rho ranging from 0.580 to 0.856, *p* < 0.01). Finally, age and years of experience were highly correlated with each other (Rho = 0.615, *p* < 0.01).

In order to conduct a differential study, the following section presents the correlational analysis between the SAS-2 factors, number of injuries, age, and years of experience for the subsamples of Alevin, Infantil, and Cadete players.

For the Alevin players, the results shown in Table 4 indicate no significant relationships between the SAS-2 factors, number of injuries, age, and years of experience.

The Somatic Anxiety factor was moderately and positively associated with Concentration Disruption (Rho = 0.334, *p* < 0.01) and with Total Anxiety (Rho = 0.501, *p* < 0.01).

The Worry factor showed a strong correlation with Total Anxiety (Rho = 0.813, *p* < 0.01) and a moderate correlation with Concentration Disruption (Rho = 0.329, *p* < 0.01). In addition, significant negative correlations were found with years of experience (Rho = −0.235, *p* < 0.05) and with age (Rho = −0.245, *p* < 0.05).

Regarding the Concentration Disruption factor, a strong association was identified with Total Anxiety (Rho = 0.716, *p* < 0.01), as well as a trend-level negative correlation with years of experience (Rho = −0.220, † *p* < 0.10) and a significant negative correlation with age (Rho = −0.324, *p* < 0.05).

As expected, the Total Anxiety factor showed strong correlations with all other SAS-2 factors, as well as significant negative correlations with years of experience (Rho = −0.264, *p* < 0.05) and with age (Rho = −0.345, *p* < 0.05).

Regarding the Infantil category, Table 5 presents the results of the correlations by age group. The number of injuries was not associated with any SAS-2 factor, although it did show trend-level positive correlations with both years of experience (Tau-b = 0.128, *p* < 0.10) and age (Tau-b = 0.129, *p* < 0.10).

The Somatic Anxiety factor was positively correlated with Worry (Rho = 0.357, *p* < 0.01) and Concentration Disruption (Rho = 0.315, *p* < 0.01) and showed a strong correlation with Total Anxiety (Rho = 0.657, *p* < 0.01).

The Worry factor was strongly associated with Total Anxiety (Rho = 0.870, *p* < 0.01). In turn, Concentration Disruption showed a moderate correlation with Total Anxiety (Rho = 0.467, *p* < 0.01).

Finally, a strong and significant correlation was observed between age and years of experience (Rho = 0.639, *p* < 0.01).

In the Cadete category, as shown in Table 6, the number of injuries was positively correlated with Somatic Anxiety (Tau-b = 0.139, *p* = 0.050) and Concentration Disruption (Tau-b = 0.146, *p* = 0.037).

Additionally, Somatic Anxiety showed significant positive correlations with Worry (Rho = 0.345, *p* < 0.001), Concentration Disruption (Rho = 0.432, *p* < 0.001), and Total Anxiety (Rho = 0.633, *p* < 0.001).

Worry was significantly correlated with Concentration Disruption (Rho = 0.359, *p* < 0.001) and very strongly with Total Anxiety (Rho = 0.886, *p* < 0.001).

As for Concentration Disruption, it was significantly associated with Total Anxiety (Rho = 0.650, *p* < 0.001).

Finally, age was significantly and positively correlated with years of experience (Rho = 0.615, *p* < 0.001).

## 4. Discussion

The present study confirms that at least 65% of young football players in the analysed sample sustain a sports injury per season, a figure consistent with previous research reporting a high incidence of injury in this population [9]. This rate is concerning, as injuries, particularly severe ones, can negatively affect the psychological well-being, physical health, and athletic development of young athletes [7,8,9]. As noted in the literature, such vulnerability arises from a multifactorial interplay of physical, psychosocial, and contextual factors within the sporting environment [14,15,16], underscoring the need for a comprehensive approach to injury prevention. This is consistent with recent international findings that stress the integration of psychological screening into injury prevention strategies for youth athletes [54].

In this regard, it has been observed that as players progress through age-based sporting categories, the number of individuals who remain injury-free decreases, while the proportion of those sustaining two or more injuries during the season increases. This pattern may be explained by multiple interrelated factors, such as maturation-related processes, the progressive increase in training loads, the accumulation of physical and mental fatigue, self-care practices (such as warm-up and stretching routines), accidental events inherent to competition, and habits related to nutrition and rest [17,18,19,22,23,24,30,31]. In addition, variables such as competitive anxiety, injury history, sporting skill, coaching leadership style, and parental influence may also exert a significant impact on the young athlete. These findings underscore the importance of continuing research from a biopsychosocial perspective in order to understand and prevent injury in youth sport appropriately.

With regard to differences in competitive anxiety (as measured by the SAS-2) across age categories, the results indicate that Cadete and Infantil players display higher levels of Worry and lower levels of Concentration Disruption than Alevin players. As noted in previous research, age category may play a key role in the manifestation of competitive anxiety [37,39], since various personal, sporting and contextual factors may influence this construct, as previously discussed.

To interpret these findings, it is important to note that Worry scores were high (mean range across categories between 12.39 and 14.00), above the arithmetic mean and approaching the maximum value for the factor (minimum = 5, maximum = 15, mean = 7.5). Worry in young athletes may be driven by factors such as performance pressure, fear of failure, the desire for team acceptance, and developmental changes. However, its intensity and content vary by age. Cadete and Infantil players tend to worry more due to increased self-awareness, greater demands, and developmental stages characterised by heightened performance-related and social-image anxiety [28,33,39]. This finding aligns with the Stress and Injury Model proposed by Andersen and Williams [30,31], which highlights the influence of situational stressors and individual differences in anxiety responses and injury likelihood. Moreover, studies such as that by Borges et al. [36] involving young water polo players support this trend, showing an increase in competitive anxiety with age and perceived performance level. Similar developmental effects on competitive anxiety across age categories have also been identified in recent studies involving young football players [55].

Concentration Disruption scores were close to the arithmetic mean for this factor (mean range across categories between 6.96 and 7.29). This indicates a moderate level of concentration difficulties among players, with Alevin players exhibiting higher disruption due to their lower cognitive maturity, reduced emotional self-regulation, and limited attentional control.

By contrast, no significant differences were observed in Somatic Anxiety according to age category, with scores also considered moderate (mean range between 6.96 and 7.29), being close to the arithmetic mean (minimum = 5, maximum = 15, mean = 7.5). Somatic Anxiety, which encompasses physical symptoms such as heart palpitations or muscle tension, remains relatively stable across ages in youth sport, as it reflects an automatic response to stress and depends more on individual profile and context than on age itself [19,27]. Both children and adolescents may experience similar physical sensations, although their interpretation varies with cognitive development [30]. Furthermore, the overall moderate scores observed for Somatic Anxiety may limit between-group variability, hindering the detection of significant differences [36].

In relation to sports injuries across the full sample, positive correlations were found with Somatic Anxiety, sporting experience, and age, in line with previous studies [27,33,34,35,36]. These findings suggest a certain vulnerability to injury among soccer players in formative stages, a group particularly sensitive at both psychological and physical levels [1,2,30], and they partially confirm the initial hypothesis

Although the positive correlations between Somatic Anxiety and the number of injuries were of low magnitude, they are consistent with earlier studies suggesting that excessive physiological arousal may impair technical execution and motor coordination [18,19,27]. Manifestations such as muscle tension, increased heart rate, or physical discomfort may prompt rushed or poorly executed movements, thereby increasing the risk of injury [18,19].

Analyses by age category and years of experience provide additional relevant insights. Young athletes face increasing physical, psychological, and competitive demands, which, along with their accumulated training history, raise their risk of injury [2,17,56]. This is not merely a biological issue but rather a consequence of an increasingly demanding sporting context.

Furthermore, age and experience are positively associated with the Worry factor and negatively associated with Concentration Disruption. Older and more experienced athletes tend to be exposed to greater demands and pressures [28,39]. At the same time, athletes with more age or experience generally exhibit better concentration and emotional regulation [17,34].

In the subgroup analysis, no significant relationship was found between injury incidence and variables such as anxiety, age, or experience in the Alevin category [26]. This suggests that injury is not directly dependent on emotional state or length of sporting practice. Injuries at this stage may instead be more closely linked to physical or contextual factors (such as technique, training load, or type of activity).

Conversely, age and experience were negatively associated with Worry, Concentration Disruption, and overall anxiety. The younger athletes’ limited experience, lower anticipatory capacity, underdeveloped abstract thinking, and emerging social judgement mean they are less likely to internalise external pressure or performance evaluation at this stage [2,4]. This lower anxiety may represent a favourable developmental window in which to consolidate technical–tactical, physical, and psychological foundations, as well as to enjoy the activity, by establishing demands and training loads adapted to this age and avoiding sources of stress or injury.

In the Infantil category, a slight increase in injury incidence was observed as players grew older and accumulated more experience, probably due to maturation-related changes and the progressive rise in training load over time [38].

However, no significant relationship was found between anxiety and injury, nor between anxiety and age or experience, suggesting that anxiety does not directly influence injury occurrence, nor is it associated with the duration of sporting involvement or age [33].

In the Cadete category, the number of injuries was positively associated with higher levels of Somatic Anxiety and Concentration Disruption. This suggests that adolescent players experiencing greater physical demand or who have greater difficulty concentrating tend to report more injuries [17]. Therefore, the way in which these athletes experience sport emotionally and cognitively appears to be related to their injury risk. This may be attributable to external factors such as increasing sporting demands, higher training loads, parental expectations, and competitive pressure [14].

Moreover, no relationship was found in this group between anxiety and variables such as age or experience, indicating that, among Cadete players, being older or having more years of practice does not necessarily influence anxiety levels.

These findings are consistent with evidence highlighting the role of psychological variables, particularly competitive anxiety and sport-related stress, as significant predictors of injury vulnerability in young athletes [21,29,35]. In the sample analysed, this relationship should be understood in the context of progressive maturation and the developmental trajectory typical of these age groups, in which physical, cognitive, and emotional dimensions are evolving simultaneously. As young athletes advance through developmental stages, they experience hormonal changes, disruptions in motor coordination, and modifications in body composition that may increase physical susceptibility. In parallel, increased sporting experience entails greater training loads, competitive pressure, self-imposed expectations, and exposure to external evaluation, all of which may elevate perceived stress and compromise emotional self-regulation. This interaction between biological maturation and the demands of the sporting environment reinforces the idea that injury risk cannot be attributed solely to physical factors but must instead be approached from a holistic perspective encompassing psychological development, stress management, and emotional support, particularly during sensitive periods of growth [16].

In the same vein, the findings support the implementation of psychological intervention programmes incorporating coping strategies, attentional skills training, relaxation techniques, and mindfulness, with the aim of reducing competitive anxiety and consequently lowering injury risk [36,37,38,39,41,42,43]. In this respect, Gil-Caselles [6] highlights the importance of including targeted psychological interventions for young athletes to promote mental health and prevent injury, thus supporting a preventive and integrative approach to youth sport.

The present study emphasises the importance of designing preventive strategies and psychological training programmes adapted to the maturational and competitive profile of young soccer players. The early identification of at-risk profiles, through instruments such as SAS-2, enables intervention not only on anxiety and its manifestations but also to promote sustained participation in sport and the overall well-being of the athlete [20,34,38].

Finally, the results provide empirical evidence supporting the inclusion of sport psychology professionals within coaching and medical teams, supporting a holistic approach to injury prevention and psychological support [17,18,20]. The observed relationship between psychological variables and injury, although moderate in some cases, underlines the interdependence of mental and physical health, both of which should be addressed together [16,29,35].

## 5. Conclusions

The results of the present study reinforce the multifactorial complexity of sports injuries in youth soccer, highlighting how psychological, maturational, and sporting variables intertwine throughout players’ development. A high incidence of injuries was confirmed in this population, particularly as players progress through sport age categories, suggesting a cumulative effect of factors such as training load, physical maturation, and the competitive environment.

The findings show that sport age influences levels of competitive anxiety. Cadete and Infantil players displayed higher levels of Worry, possibly due to increased psychological, social, and competitive demands, while Alevin players reported greater Concentration Disruption, likely associated with lower cognitive maturity. No significant differences were found in Somatic Anxiety, indicating a relatively stable physiological response across categories. These results emphasise the need for developmentally appropriate interventions to address anxiety effectively in youth sport.

Overall, sports injuries were positively associated with Somatic Anxiety, reflecting greater injury vulnerability among developing soccer players. Although the correlations were small, the findings support the idea that excessive physiological arousal may impair performance and increase injury risk. Moreover, older and more experienced players sustained a higher number of injuries and showed greater Worry but lower Concentration Disruption, suggesting increasing competitive demands alongside improved emotional regulation.

By contrast, in the Alevin category, no significant associations were found between injuries and psychological or experience-related variables, suggesting that injuries at these ages may depend more on physical or contextual factors. In addition, Alevin players reported lower levels of Worry and overall anxiety, likely due to their limited cognitive development and reduced exposure to competitive pressure. This stage represents a key opportunity to foster enjoyment, technical–tactical learning, and appropriate training loads without generating unnecessary stress.

In the Infantil category, a slight increase in injury incidence was observed with age and experience, possibly due to maturation and increased training demands. However, no significant associations were found between anxiety and injuries, nor between anxiety and age or experience, suggesting that anxiety does not directly influence injury risk at this stage.

In the Cadete category, the number of injuries was positively associated with Somatic Anxiety and Concentration Disruption, indicating that athletes with higher physiological arousal and attentional difficulties are more prone to injury. These findings suggest that emotional and mental states may influence injury risk, potentially in response to increased external demands. No associations were found between anxiety and age or experience, suggesting these factors do not directly determine anxiety levels at this stage.

In conclusion, these findings are consistent with the Andersen and Williams stress–injury model, highlighting the importance of addressing injury vulnerability from a biopsychosocial perspective. Interactions among physical maturation, competitive pressure, emotional development, training loads, mental fatigue, and leadership styles should be taken into account when designing injury prevention strategies.

It is recommended to implement psychological intervention programmes tailored to the maturational and competitive levels of youth soccer players, incorporating emotional regulation strategies, attentional training, and stress management techniques. Likewise, the systematic use of tools such as SAS-2 in medical and sports protocols could facilitate the early identification of at-risk profiles and enhance prevention efforts. These findings reinforce the need to integrate sport psychology professionals into coaching and medical teams, promoting a preventive, multidisciplinary approach that considers both physical and mental well-being as key pillars of performance and athletic development.

## 6. Limitations and Future Lines of Research

While this study offers valuable insights into the relationship between competitive anxiety and sports injuries in young soccer players, several limitations must be acknowledged. Firstly, the sample consisted exclusively of male athletes from a single club, which restricts the generalisability of the findings to other populations, such as female players or athletes from different competitive levels and sociocultural contexts. Future research should therefore include more diverse and representative samples to explore possible gender- and context-related differences.

Another limitation is the use of a cross-sectional design and the exclusive assessment of the trait dimension of competitive anxiety at a single time point, without considering state or situational anxiety. This limits the understanding of how emotional responses may vary across the season. Longitudinal studies could provide a more dynamic view of the evolution of anxiety and its association with injury incidence.

Furthermore, the study did not differentiate between types or severity of injuries, nor did it classify players based on the number of injuries sustained. This hinders the interpretation of how injury characteristics might influence psychological outcomes. Future research could incorporate more detailed injury data and profile analysis to address this gap.

The sole reliance on self-report questionnaires also presents a limitation, given their susceptibility to social desirability bias and subjective interpretation. To enhance validity, future studies might integrate psychophysiological measures or observational tools as complementary methods.

Finally, the study did not include other potentially relevant psychological or contextual variables, such as resilience, coping strategies, motivational climate, or external stressors (e.g., academic or family-related). These factors could mediate or moderate the relationship between anxiety and injury. It is therefore recommended that future research adopt a multivariable and interdisciplinary approach that incorporates psychological, physical, and contextual components, in line with the Stress and Injury Model proposed by Andersen and Williams.

In addition, further studies could evaluate the effectiveness of psychological intervention programmes, including mindfulness, relaxation techniques, and psychological skills training, aimed at reducing competitive anxiety and preventing injuries. This holistic perspective would not only broaden scientific understanding but also support practical applications in the field of youth sports.

## 7. Practical Applications

The findings of this study have relevant implications for coaches, support staff, sport psychologists, and other professionals involved in the holistic development of young soccer players. The identification of an increase in injury incidence with age and the influence of psychological variables such as anxiety underscores the need to design intervention programmes tailored to the athletes’ developmental and competitive stages. These programmes should integrate psychological training, including coping strategies, relaxation techniques, attentional control, and goal setting with the aim of reducing competitive anxiety and decreasing injury vulnerability.

Furthermore, it is essential to consider these aspects within a coherent sporting management approach, where the coach’s leadership style plays a key role. Promoting a positive leadership style based on emotional support, effective communication, and reinforcement of effort rather than outcome contributes to the development of mastery-oriented motivational climates that foster enjoyment while minimising excessive pressure, social comparison, and fear of failure.

From a training design perspective, it is advisable to adapt the workload and dynamics to the players’ maturational characteristics, progressively incorporating elements of emotional self-regulation and decision-making under pressure, as well as opportunities for reflection and constructive feedback.

In addition, regular assessment of psychological variables (such as competitive anxiety, perceived stress, or emotional well-being) is recommended as part of the physical and medical protocols. This would facilitate a preventive, interdisciplinary, and athlete-centred approach. Such monitoring would enable the early identification of psychological risk profiles and the implementation of timely, personalised interventions to prevent injury and promote overall well-being.

Finally, it is crucial to raise awareness among families and sports stakeholders about the impact of competitive anxiety on health and performance, encouraging a healthier and more educational sporting culture focused on the young athlete’s personal development beyond competitive outcomes.

## Figures and Tables

**Table 1:** Contingency table considering sport category and injury frequencies.

Number of Injuries	0	1	2	3	4	5	6	8
Alevin (*n* = 72)								
Count	30	25	14	1	0	0	1	1
% of category	41.7%	34.7%	19.4%	1.4%	0.0%	0.0%	1.4%	1.4%
Infantil (*n* = 115)								
Count	41	36	28	8	2	0	0	0
% of category	35.7%	31.3%	24.3%	7.0%	1.7%	0.0%	0.0%	0.0%
Cadete (*n* = 135)								
Count	37	45	34	9	7	2	1	0
% of category	27.4%	33.3%	25.2%	6.7%	5.2%	1.5%	0.7%	0.0%
Total (*n* = 322)								
Count	108	106	76	18	9	2	2	1
% of total	33.5%	32.9%	23.6%	5.6%	2.8%	0.6%	0.6%	0.3%

Note. *n* = number of players; % = percentage within each sport category.

**Table 2:** Descriptive statistics, mean difference analysis, and effect size (Hedges’ g) of the SAS-2 questionnaire according to sport age category.

Variable	Group	*N*	*M*	*SD*	Kruskal–Wallis			Pairwise Mann–Whitney U				Hedges Ĝ
					Mean Rank	Chi-Square	Sig.	Pairs	U	z	Sig.	
SAS—Somatic	Alevin	72	7.07	2.00	167.42	2.551	0.279	B–I	4103.500	−0.10	0.918	0.091
	Infantil	115	7.29	2.57	168.94			B–C	4397.500	−1.15	0.247	0.046
	Cadete	135	6.96	2.65	152.01			I–C	6943.500	−1.47	0.140	0.126
	Total	322	7.10	2.49								
SAS—Worry	Alevin	72	12.39	3.84	133.06	8.717	0.013 *	B–I *	3226.500	−2.54	0.011 *	0.380
	Infantil	115	13.92	4.11	168.86			B–C **	3726.000	−2.77	0.006 **	0.420
	Cadete	135	14.00	3.81	170.40			I–C	7695.500	−0.11	0.906	0.020
	Total	322	13.61	3.97								
SAS—Distractibility	Alevin	72	7.94	2.13	199.32	16.300	0.000 ***	B–I ***	2983.500	−3.26	0.001 ***	0.508
	Infantil	115	6.96	1.80	154.82			B–C ***	3293.500	−3.88	0.000 ***	0.477
	Cadete	135	6.90	2.19	147.02			I–C	7374.500	−0.69	0.487	0.026
	Total	322	7.16	2.08								
SAS—Total	Alevin	72	27.40	5.72	159.31	0.350	0.839	B–I	3982.500	−0.43	0.661	0.123
	Infantil	115	28.17	6.41	165.61			B–C	4860.000	0.00	1.000	0.070
	Cadete	135	27.86	6.82	159.16			I–C	7447.000	−0.55	0.579	0.046
	Total	322	27.87	6.43								

* *p* < 0.05, ** *p* < 0.01, *** *p* < 0.001; *N* = sample size; *M* = mean; *SD* = standard deviation.

**Table 3:** Correlational analysis using Spearman’s Rho and Kendall’s Tau-b coefficients (only crossings with number of injuries; total sample; *n* = 322) for the total sample between SAS-2 factors, SAS total score, number of injuries, years of training, and age.

	SAS—Somatic	SAS—Worry	SAS—Distractibility	SAS—Total	Number of Injuries	Years of Experience	Age
SAS—Somatic	1.000	0.281 **	0.380 **	0.611 **	0.091 *	−0.080	−0.053
		<0.001	<0.001	<0.001	0.045	0.154	0.340
SAS—Worry		1.000	0.225 **	0.856 **	0.000	0.094 †	0.140 *
			<0.001	<0.001	0.991	0.093	0.012
SAS—Distractibility			1.000	0.580 **	0.029	−0.200 **	−0.229 **
				<0.001	0.522	<0.001	<0.001
SAS—Total				1.000	0.023	−0.037	−0.009
					0.592	0.509	0.873
Number of injuries					1.000	0.135 **	0.146 **
						0.002	0.001
Years of experience						1.000	0.615 **
							<0.001
Age							1.000

Notes: † *p* < 0.10; * *p* < 0.05; ** *p* < 0.01

**Table 4:** Correlational analysis using Spearman’s Rho and Kendall’s Tau-b coefficients (only crossings with number of injuries; Alevin sample; *n* = 72) between SAS factors, SAS-2 total score, number of injuries, and years of training.

	SAS—Somatic	SAS—Worry	SAS—Distractibility	SAS—Total	Number of Injuries	Years of Experience	Age
SAS—Somatic	1.000	0.089	0.334 **	0.501 **	0.041	−0.116	−0.144
		0.456	0.004	<0.001	0.679	0.333	0.455
SAS—Worry		1.000	0.329 **	0.813 **	−0.123	−0.235 *	−0.245 *
			0.005	<0.001	0.192	0.047	0.035
SAS—Distractibility			1.000	0.716 **	0.063	−0.220 †	−0.324 *
				<0.001	0.514	0.064	0.025
SAS—Total				1.000	−0.071	−0.264 *	−0.345 *
					0.447	0.025	0.015
Number of injuries					1.000	0.062	0.089
						0.531	0.455
Years of experience						1.000	0.565 *
							<0.001
Age							1.000

Notes: † *p* < 0.10; *p < 0.05; **p < 0.01

**Table 5:** Correlational analysis using Spearman’s Rho and Kendall’s Tau-b coefficients (only crossings with number of injuries; Infantil sample; *n* = 115) between SAS factors, SAS-2 total score, number of injuries, and years of training.

	SAS—Somatic	SAS—Worry	SAS—Distractibility	SAS—Total	Number of Injuries	Years of Experience	Age
SAS—Somatic	1.000	0.357 **	0.315 **	0.657 **	0.086	−0.130	−0.118
		<0.001	<0.001	<0.001	0.259	0.167	0.204
SAS—Worry		1.000	0.152	0.870 **	0.045	0.093	0.100
			0.105	<0.001	0.541	0.326	0.293
SAS—Distractibility			1.000	0.467 **	−0.064	0.014	−0.004
				<0.001	0.408	0.885	0.966
SAS—Total				1.000	0.031	0.023	0.038
					0.667	0.810	0.753
Number of injuries					1.000	0.128 †	0.129 †
						0.095	0.10
Years of experience						1.000	0.639 **
							*p* < 0.001
Age							1.000

Notes: † *p* < 0.10; ** *p* < 0.01

**Table 6:** Correlational analysis using Spearman’s Rho and Kendall’s Tau-b coefficients (only crossings with number of injuries; Cadet sample; n = 135) between SAS factors, SAS-2 total score, number of injuries, and years of training.

	SAS—Somatic	SAS—Worry	SAS—Distractibility	SAS—Total	Number of Injuries	Years of Experience	Age
SAS—Somatic	1.000	0.345 **	0.432 **	0.633 **	0.139 †	0.052	−0.060
		<0.001	<0.001	<0.001	0.050	0.550	0.390
SAS—Worry		1.000	0.359 **	0.886 **	−0.021	−0.010	0.008
			<0.001	<0.001	0.748	0.911	0.926
SAS—Distractibility			1.000	0.650 **	0.146 *	−0.115	−0.084
				<0.001	0.037	0.184	0.275
SAS—Total				1.000	0.065	−0.036	−0.042
					0.323	0.677	0.630
Number of injuries					1.000	0.063	0.058
						0.362	0.404
Years of experience						1.000	0.615 **
							<0.001
Age							1.000

Notes: † *p* < 0.10; * *p* < 0.05; ** *p* < 0.01

## Data Availability

The raw data supporting the conclusions of this article will be made available by the authors, without undue reservation.

## Abbreviations

The following abbreviations are used in this manuscript:

SAS-2	Sport Anxiety Scale
